# Superior mesenteric artery Doppler parameters across sepsis severity spectrum and their discriminative performance for refractory septic shock

**DOI:** 10.3389/fmed.2026.1990309

**Published:** 2026-10-27

**Authors:** Hao Pu, Shujun Zhou, Xinyi Liu, Tao Zeng, Wanru Li, Feng Ping, Siwei Wang

**Affiliations:** 1 Department of Critical Care Medicine, Sichuan Clinical Research Center for Cancer, Sichuan Cancer Hospital & Institute, Sichuan Cancer Center, School of Medicine, University of Electronic Science and Technology of China, Chengdu, China; 2 Department of Critical Care Medicine, The Third Affiliated Hospital of Soochow University, The First People's Hospital of Changzhou, Changzhou, Jiangsu, China; 3 Department of Thoracic Oncology, Sichuan Clinical Research Center for Cancer, Sichuan Cancer Hospital & Institute, Sichuan Cancer Center, School of Medicine, University of Electronic Science and Technology of China, Chengdu, China

**Keywords:** Doppler ultrasonography, refractory shock, sepsis, septic shock, superior mesenteric artery

## Abstract

**Background:**

This study aimed to characterize the evolution of superior mesenteric artery (SMA) Doppler parameters across the sepsis-severity spectrum, evaluate their exploratory discriminative performance for refractory septic shock, and explore whether waveform characteristics change after successful resuscitation.

**Methods:**

In this single-center retrospective observational study, 96 patients were enrolled: non-septic ICU controls (*n* = 22), sepsis (*n* = 29), septic shock (*n* = 22), and refractory septic shock (*n* = 23). Bedside SMA Doppler ultrasound measured PSV, EDV, TAMV, RI, PI, and BF. SMA Doppler parameters were compared across the four groups; correlations with illness-severity markers and norepinephrine-dose-stratified analyses were performed.

Cross-sectional discrimination for refractory septic shock was assessed using Firth's penalized logistic regression and ROC analysis with bootstrap internal validation (1,000 replicates) in acute-phase shock patients. In a subgroup of 20 shock patients who achieved successful resuscitation, paired pre- and post-resuscitation measurements were obtained.

**Results:**

96 patients were enrolled. SMA hemodynamic profiles differed significantly across four acute-phase cohorts (all *p* < 0.001). Sepsis patients exhibited compensatory hyper-dynamic mesenteric features; with advancing illness towards refractory septic shock, SMA flow velocities and resistance indices declined significantly. PSV, RI and PI were inversely associated with SOFA score, lactate, norepinephrine dosage and creatinine (all *p* < 0.001). Despite stable mean arterial pressure, RI and PI decreased progressively with escalating norepinephrine dose. Among acute-phase shock patients(*n* = 45), PSV and RI showed apparent AUCs of 0.898 and 0.874, respectively; optimism-corrected AUC after bootstrap internal validation were 0.858 for PSV and 0.823 for RI. In the paired subgroup, PSV and RI increased after successful resuscitation (*p* < 0.001), whereas TAMV and calculated BF did not change significantly.

**Conclusion:**

Bedside SMA Doppler ultrasonography may capture a non-linear mesenteric hemodynamic trajectory across sepsis severity. A low-resistance, low-velocity SMA Doppler phenotype is associated with more severe shock and may serve as an exploratory discriminative marker for refractory septic shock.

## Introduction

Septic shock remains a leading cause of mortality in intensive-care units worldwide, driven by dysregulated host responses to infection and progressive multiple-organ dysfunction (1). Within this pathophysiological cascade, the gastrointestinal tract is widely recognized as the “motor” of multiple organ dysfunction syndrome (2, 3). Despite widespread implementation of resuscitation bundles, substantial numbers of patients progress to refractory septic shock characterized by profound circulatory collapse, escalating vasopressor requirements and high mortality (4, 5).

A core clinical dilemma is hemodynamic incoherence: macro-circulatory goals such as mean arterial pressure may be restored by resuscitation, while regional splanchnic perfusion continues to deteriorate (6). Conventional systemic markers, including lactate and central venous oxygen saturation (ScvO₂), reflect global metabolic status and often lag behind microvascular failure in specific organ beds (7, 8). By the time these systemic indicators signal refractory shock, irreversible ischemic injury and intestinal mucosal barrier breakdown may already have occurred (9). Hence, non-invasive real-time biomarkers reflecting splanchnic perfusion are clinically desirable.

Bedside superior mesenteric artery (SMA) Doppler ultrasound enables non-invasive assessment of visceral mesenteric hemodynamics. Prior studies have reported altered SMA flow patterns in critically-ill patients (10, 11). However, sequential changes in SMA Doppler indices across the full clinical spectrum ranging from uncomplicated sepsis to refractory septic shock remain incompletely described. Specifically, the hypothesis that mesenteric circulation shifts from compensatory vasoconstriction in early sepsis towards a low-resistance phenotype in refractory shock lacks systematic clinical evidence (12, 13). Moreover, it remains unclear whether mesenteric hemodynamic disturbances can reverse once resuscitation endpoints are achieved.

This retrospective observational study aimed to characterize acute-phase SMA-Doppler profiles across cohorts of escalating sepsis severity, assess their exploratory discriminative value for refractory septic shock, and explore whether SMA Doppler waveform characteristics change after successful resuscitation using paired pre- and post-resuscitation measurements in a subset of patients.

## Methods

### Study design and population

This was a retrospective analysis of prospectively collected data conducted in the Department of Critical Care Medicine at the Third Affiliated Hospital of Soochow University from January 2023 to August 2023. The study protocol was designed in accordance with the Declaration of Helsinki and received formal approval from the Institutional Review Board. Written informed consent was obtained from all patients or their legally authorized representatives.

### Participant selection and group definitions

A total of 96 participants were enrolled and stratified into four groups based on international consensus criteria and clinical trajectories:

• Non-septic ICU Controls (*n* = 22): Patients admitted to the Intensive Care Unit (ICU) for non-septic conditions (e.g., postoperative monitoring, non-infectious trauma or acute cerebrovascular disease) who exhibited hemodynamic stability.
• Sepsis (*n* = 29): Defined according to the Sepsis-3 criteria as infection-induced organ dysfunction, characterized by an acute increase in the Sequential Organ Failure Assessment (SOFA) score of ≥ 2 points (1).
• Septic Shock (*n* = 22): Defined as sepsis requiring vasopressors to maintain a mean arterial pressure (MAP) ≥ 65mmHg and a serum lactate level > 2.0 mmol/L despite adequate fluid resuscitation (1).
• Refractory Septic Shock (*n* = 23): In accordance with the SCCM/ESICM Delphi consensus, defined as septic shock with persistent hyperlactatemia and/or persistently prolonged capillary refill time (CRT) after adequate initial fluid resuscitation, confirmed fluid non-responsiveness, and a norepinephrine-equivalent dose (NEE) > 0.5 μg/kg/min (14).
1. Persistent hyperlactatemia was defined as arterial lactate ≥2 mmol/L on two consecutive measurements separated by at least 2 h after adequate fluid resuscitation.
2. CRT was measured by applying pressure to the ventral surface of the distal phalanx (fingertip) of the index finger for 10 s, and recording the time to return to normal skin color. CRT >3 s was considered prolonged (15).
3. Fluid non-responsiveness was evaluated according to respiratory status. In mechanically ventilated patients meeting the validity criteria for pulse pressure variation (PPV), PPV <12% defined fluid non-responsiveness (16). For spontaneously breathing patients, a passive leg raise (PLR) test was performed, and a rise in left ventricular outflow tract velocity-time integral (LVOT VTI) of less than 10% indicated fluid non-responsiveness (17); if PLR was inconclusive, a mini-fluid challenge (100 mL crystalloid over 1 min) was performed, with a rise in LVOT VTI of less than 5% defined as fluid non-responsiveness (18).
4. NEE was calculated as (19): NEE=norepinephrine dose+epinephrine dose+Vasopressin (U/min) × 2.5 + phenylephrine dose×0.1. Only norepinephrine and pituitrin were administered in this study; because pituitrin lacks a standardized vasopressin-equivalent conversion, it was not included in NEE calculation. No epinephrine or phenylephrine was used. Therefore, in this cohort, NEE was equivalent to the norepinephrine dose.
• Successful Resuscitation (*n* = 20): Patients, selected from the septic shock and refractory Septic Shock group, who achieved hemodynamic stability (MAP ≥ 65mmHg with minimal or no vasopressor support, defined as a norepinephrine dose of ≤ 0.2*μ*g/kg/min), metabolic restoration (lactate ≤ 2.0 mmol/L), and multi-organ functional recovery maintained for a minimum duration of 24 consecutive hours after comprehensive treatment.

Exclusion Criteria: Age < 18 years; pregnancy; primary gastrointestinal disease (e.g., bowel obstruction, mesenteric ischemia); abdominal infections; post-operative from abdominal surgery; or poor ultrasound image quality of SMA.

### Norepinephrine dose subgroup definitions

For the dose-response analysis, patients receiving norepinephrine support were further stratified into three subgroups according to concurrent norepinephrine equivalent (base) dose obtained at the exact time point of SMA Doppler examination: (1) Low-dose group: ≤ 0.2μg/kg/min; (2) Moderate-dose group: > 0.2 to ≤ 0.5μg/kg/min; (3) High-dose group: > 0.5μg/kg/min.

### SMA Doppler ultrasonography measurement

Bedside Doppler ultrasound of the superior mesenteric artery (SMA) was performed by two experienced intensivists using the Konica Sonimage HS1 system with a 1–5 MHz convex transducer. Measurements were obtained in the supine position, at end-expiration, in the fasting state. The SMA was visualized 1–2 cm distal to its aortic origin, with pulsed-wave Doppler at an insonation angle < 60°. Peak systolic velocity (PSV), end-diastolic velocity (EDV), and time-averaged mean velocity (TAMV) were averaged over three consecutive cardiac cycles. Vessel diameter (D) was measured at its proximal segment (1–2 cm distal to the origin from the abdominal aorta) in a B-mode longitudinal or transverse view at end-diastole using the inner-edge-to-inner-edge technique. The cross-sectional area (CSA) was determined as $CSA=\pi \;\times D^{2}/4.$ The resistive index (RI) was calculated using the formula: RI=(PSV−EDV)/PSV. Similarly, the pulsatility index (PI) was calculated using the formula: PI=(PSV−EDV)/TAMV. The blood flow was calculated using the formula: $\mathrm{BF}\;=\;\pi \times D^{2}/4\;\times \mathrm{TAMV}\times 60.$ All measurements in sepsis, septic shock, and refractory septic shock groups were obtained within 2 h of meeting diagnostic criteria.

### Clinical data collection

Baseline characteristics, including age, sex, and body mass index (BMI), were documented upon enrollment. Disease severity was assessed using the Acute Physiology and Chronic Health Evaluation II (APACHE II) and Sequential Organ Failure Assessment (SOFA) scores. Concomitantly with the superior mesenteric artery (SMA) ultrasound, real-time macro-hemodynamic parameters and systemic perfusion markers—comprising heart rate (HR), mean arterial pressure (MAP), norepinephrine infusion rate at the time of examination, arterial blood lactate (Lac), and venous-to-arterial carbon dioxide tension difference (Pv-aCO_2_)—were recorded to ensure hemodynamic coherence. Additionally, a comprehensive biochemical panel was analyzed to evaluate inflammatory response and organ function, including white blood cell (WBC) count, C-reactive protein (CRP), procalcitonin (PCT), total bilirubin, serum creatinine (Scr), and cardiac troponin I (cTnI).

### Statistical analysis

Normality was assessed using the Shapiro–Wilk test. Continuous variables are presented as mean ± SD or median (IQR); categorical variables as counts (%). The four acute-phase groups were independent and compared using one-way analysis of variance (ANOVA) with Tukey *post hoc* test or the Kruskal–Wallis H test with Dunn–Bonferroni correction, as appropriate. Categorical data were compared using the chi-squared or Fisher's exact test. Spearman's rank-order correlation was used to evaluate associations between SMA Doppler parameters and severity markers; correlation coefficients and corresponding *p*-values are reported, with statistical significance also indicated by asterisks in Figure 4. For discriminative performance analyses within the shock cohort (septic shock+refractory septic shock, *n* = 45), Firth's penalized logistic regression was used to mitigate small-sample bias. Variables with *p* < 0.05 in univariable screening were assessed for multicollinearity, with variance inflation factor (VIF) ≥ 5 indicating collinearity. PSV and RI were incorporated into the final multivariable model. Discriminative performance was assessed using receiver operating characteristic (ROC) curve analysis and the area under the curve (AUC). The apparent AUCs and their 95% confidence interval (CI) were estimated via the nonparametric asymptotic method. Bootstrap resampling with 1,000 replicates was performed to obtain the optimism-corrected AUCs and their 95% CIs. Cut-off values were determined by maximizing the Youden index. At these fixed cut-off values, sensitivity and specificity were calculated, with 95% CI derived from bootstrap resampling. For the paired successful-resuscitation subgroup (*n* = 20), paired-sample analyses compared SMA Doppler parameters at acute shock baseline and ≥24 h after meeting predefined resuscitation endpoints. Within-patient differences were assessed for normality using the Shapiro–Wilk test. Paired *t*-tests were used for normally distributed differences, with data presented as mean ± SD. Wilcoxon signed-rank tests were used for non-normally distributed differences, with data presented as median (IQR). Individual-level changes were visualized using spaghetti plots. Analyses were performed using SPSS 26.0 and R 4.3.2. A two-tailed *p* < 0.05 was considered significant.

## Results

### Participants and baseline characteristics

A total of 164 patients were initially assessed for eligibility. After excluding 68 patients, 96 patients were finally enrolled in this study **(**Figure 1). Baseline characteristics are summarized in Table 1. Age, sex, and BMI were comparable across the four groups (all *p* > 0.05). Illness severity scores (APACHE II, SOFA) increased progressively from controls to refractory septic shock (both *p* < 0.001), paralleled by significant elevations in inflammatory markers (WBC, PCT, CRP), organ dysfunction biomarkers (creatinine, troponin I), and perfusion indices (lactate, Pv-aCO₂; all *p* < 0.001). While mean arterial pressure was preserved (*p* = 0.175), norepinephrine requirements, CRRT utilization, and 28-day mortality rose significantly with shock severity (all *p* < 0.001). Patients with successful resuscitation showed restored metabolic parameters and reduced vasopressor requirements compared with the refractory shock group.

**Figure 1:**
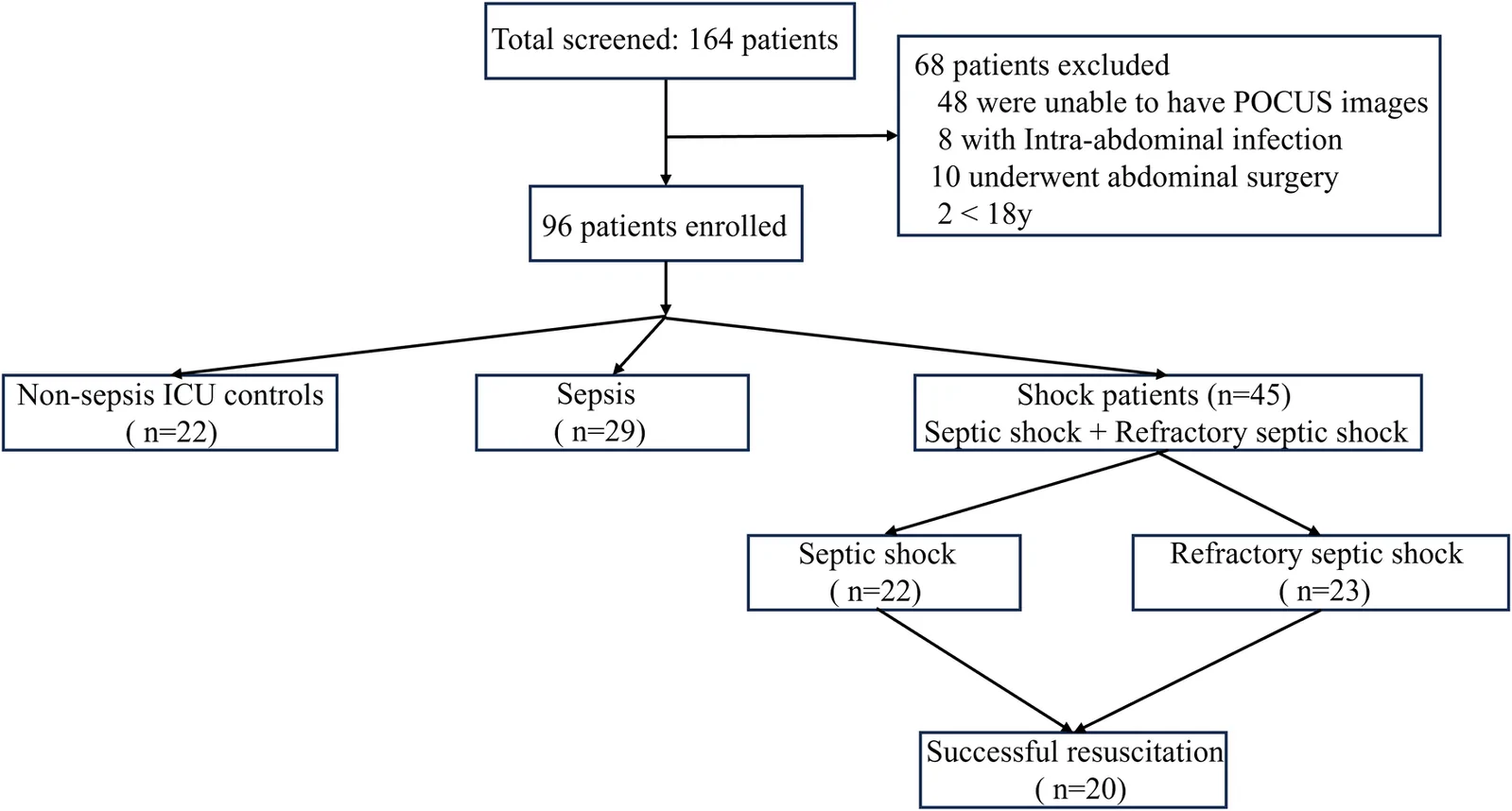
Flowchart of patient enrollment. POCUS: point-of-care ultrasound.

**Table 1:** Baseline characteristics of the study population.

Characteristic	Control (*n* = 22)	Sepsis (*n* = 29)	Septic shock (*n* = 22)	Refractory septic shock (*n* = 23)	*P* Value
Age (years)	71.00 (52.00, 78.00)	76.00 (67.00, 84.00)	75.00 (65.000, 82.00)	75.00 (63.00, 81.00)	0.328
Male Sex *n* (%)	12 (54.54)	15 (51.72)	11 (50.00)	13 (56.52)	0.757
BMI	22.49 (20.76, 25.53)	22.14 (19.37, 24.91)	22.88 (19.38, 25.95)	22.38 (19.11, 25.46)	0.851
APACHE II	20.00 (15.00, 26.00)	23.00 (18.00, 27.00)	29.00 (23.00, 33.00)	30.00 (27.00, 34.00)	<0.001
SOFA score	2.00 (1.00, 3.00)	5.00 (4.00, 7.00)	8.00 (6.00, 10.00)	12.00 (10.00, 15.00)	<0.001
WBC (×10^9^/L)	11.84 (8.60, 13.10)	12.24 (7.59, 16.17)	17.68 (14.75, 28.00)	18.93 (15.44, 30.00)	<0.001
Hemoglobin (g/L)	111.00 (99.00, 122.00)	101.00 (82.00, 113.00)	102.00 (91.00, 115.00)	100.00 (74.00, 120.00)	0.239
TB (μmol/L)	15.45 (11.70, 26.10)	17.30 (10.65, 27.75)	22.10 (12.30, 30.87)	24.10 (15.37, 37.95)	0.565
Cr (μmol/L)	72.00 (52.00, 113.00)	86.00 (55.00, 128.00)	149.00 (107.00, 206.00)	156.00 (120.00, 238.00)	<0.001
CRP (mg/L)	55.00 (19.00, 109.00)	117.00 (69.00, 185.00)	121.85 (51.00, 163.00)	152.00 (105.00, 208.00)	0.004
PCT (ng/mL)	0.30 (0.25, 0.60)	1.50 (0.29, 9.25)	16.54 (2.69, 55.36)	21.92 (6.10, 87.85)	<0.001
cTnI (ng/ml)	0.03 (0.01, 0.20)	0.24 (0.15, 0.53)	0.46 (0.09, 0.88)	0.94 (0.42, 2.60)	<0.001
Lactate (mmol/L)	1.05 (1.00, 1.30)	2.20 (1.20, 2.80)	4.25 (2.70, 7.10)	6.05 (4.45, 11.00)	<0.001
Pv-aCO_2_(mmHg)	5.00 (4.00, 6.00)	6.00 (4.00, 6.00)	8.00 (6.00, 9.00)	10.00 (7.00, 12.00)	<0.001
MAP (mmHg)	88.00 (80.00, 98.00)	87.00 (79.00, 94.00)	82.00 (68.00, 96.00)	80.00 (63.00, 93.00)	0.175
HR (bpm)	86.00 (70.00, 95.00)	96.00 (83.00, 114.00)	106.00 (92.00, 118.00)	110.00 (88.00, 127.00)	0.002
NE (μg/kg/min)	00.00 (0.00, 0.00)	0.10 (0.00, 0.20)	0.50 (0.30, 0.85)	0.75 (0.50, 1.30)	<0.001
CRRT use *n* (%)	1 (4.5)	3 (10.34)	10 (45.45)	13 (56.52)	<0.001
28 d mortality	2 (9.09)	7 (24.14)	10 (45.45)	14 (60.87)	<0.001

Data are presented as median (interquartile range) or *n* (%). Group comparisons were performed using the Kruskal–Wallis test for continuous variables and the *χ*² test or Fisher's exact test for categorical variables. APACHE II, Acute Physiology and Chronic Health Evaluation II; SOFA, Sequential Organ Failure Assessment; BMI, body mass index; WBC, white blood cells; TB, total bilirubin; Cr, Creatinine; CRP, C-reactive protein; PCT, procalcitonin; cTnI, cardiac troponin I; Pv-aCO_2_, central venous-to-arterial carbon dioxide difference; MAP, mean arterial pressure; HR, heart rate; NE, norepinephrine; CRRT, continuous renal replacement therapy.

### Comparisons of SMA Doppler parameters among groups

Spectral Doppler analysis demonstrated significant hemodynamic variations across the four cohorts (Table 2; Figures 2, 3). Qualitatively, SMA waveforms evolved from a high-resistance profile in controls and a hyperdynamic pattern in sepsis to a broadened spectrum in septic shock and a flattened, low-resistance phenotype in refractory septic shock. Quantitatively, PSV peaked in sepsis (188.20 cm/s) but fell markedly in refractory septic shock (69.00 cm/s; *P* < 0.001). EDV peaked in septic shock (27.00 cm/s; *P* < 0.001), whereas RI, PI, and BF progressively declined with shock severity and were lowest in refractory septic shock (all *P* < 0.001).

**Table 2:** Comparison of SMA Doppler parameters among four groups.

Variables	Control (*n* = 22)	Sepsis (*n* = 29)	Septic Shock (*n* = 22)	Refractory septic shock (*n* = 23)	*P* Value
PSV (cm/s)	115.40 (98.90, 136.90)	188.20 (173.70, 210.55)	111.70 (100.84, 128.63)	69.00 (63.22, 88.00)	<0.001
EDV (cm/s)	19.90 (16.37, 21.05)	21.40 (15.75, 25.85)	27.00 (22.7, 30.15)	19.65 (16.03, 25.02)	<0.001
RI	0.83 (0.82, 0.85)	0.88 (0.86, 0.92)	0.77 (0.73, 0.79)	0.72 (0.63, 0.76)	<0.001
PI	2.39 (2.09, 2.68)	2.97 (2.52, 3.51)	1.77 (1.60, 1.94)	1.50 (1.11, 1.79)	<0.001
TAMV (cm/s)	17.65 (15.42, 19.95)	24.60 (19.75, 29.10)	20.15 (17.32, 22.80)	13.55 (11.10, 17.05)	<0.001
BF (ml/min)	368.16 (306.60, 473.13)	450.24 (321.12, 588.96)	375.69 (303.39, 443.58)	264.00 (201.55, 318.54)	<0.001
CSA (cm^2^)	0.33 (0.27, 0.39)	0.30 (0.25, 0.35)	0.31 (0.26, 0.36)	0.32(0.25, 0.39)	0.681

Data are presented as median (interquartile range). Group comparisons were performed using the Kruskal–Wallis test. PSV, peak systolic velocity; EDV, end-diastolic velocity; TAMV, time-averaged mean velocity; RI, resistive index; PI, pulsatility index; BF, blood flow; CSA, cross-sectional area.

**Figure 2:**
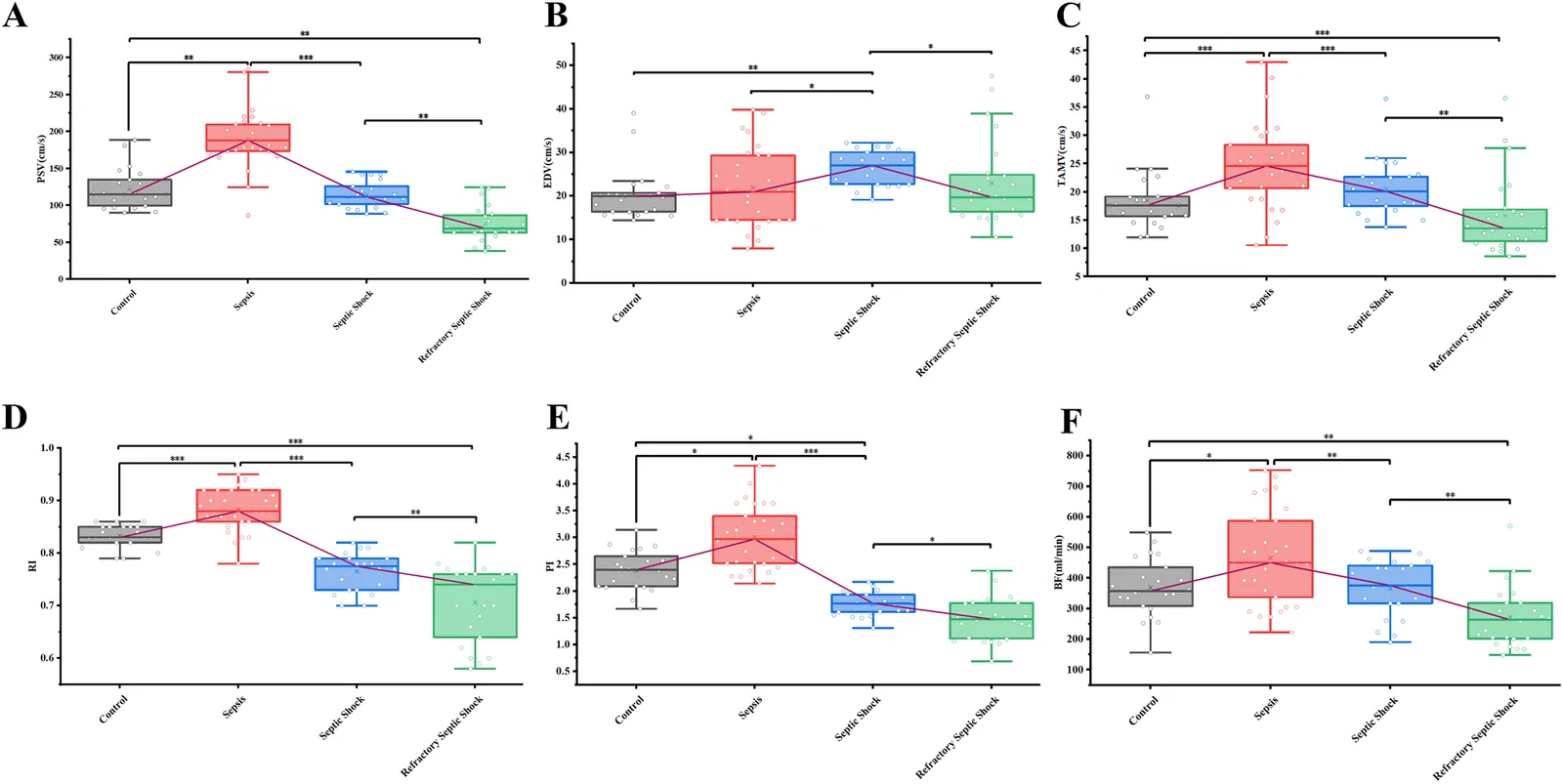
Comparison of SMA Doppler parameters among four study groups. Box-and-whisker plots illustrating **(A)** peak systolic velocity (PSV), **(B)** end-diastolic velocity (EDV), **(C)** time-averaged mean velocity (TAMV), **(D)** resistive index (RI), **(E)** pulsatility index (PI), **(F)** blood flow (BF) in non-septic ICU control(*n* = 22), sepsis(*n* = 29), septic shock(*n* = 22), and refractory septic shock(*n* = 23). The Kruskal–Wallis test followed by Dunn's *post-hoc* test with correction for multiple comparisons was used. *: *p* < 0.05; **: *p* < 0.01; ***: *p* < 0.001.

**Figure 3:**
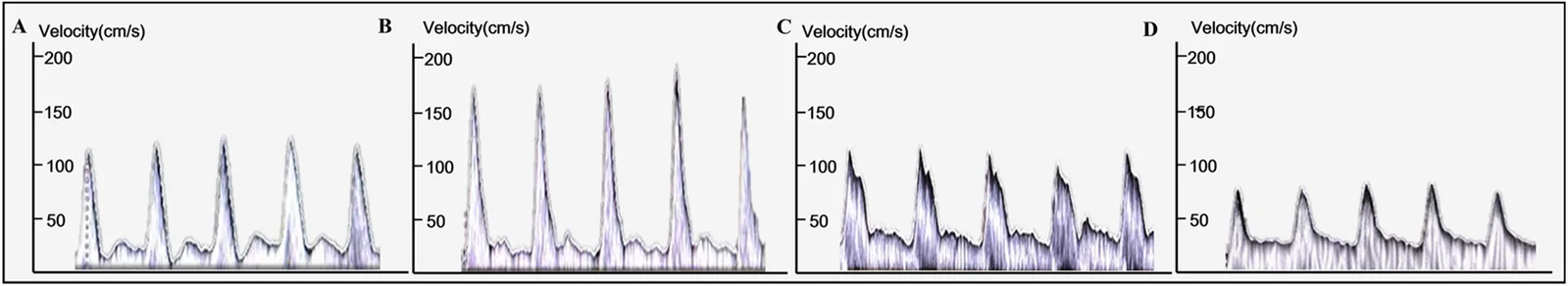
Representative SMA spectral Doppler waveforms across sepsis severity groups (0–200 cm/s velocity scale). **(A)** Control: Normal high-resistance profile with a sharp systolic peak and minimal end-diastolic flow. **(B)** Sepsis: Hyperdynamic pattern with markedly elevated peak systolic velocity and high resistive index. **(C)** Septic shock: Waveform broadening with reduced peak velocity and increased diastolic flow. **(D)** Refractory septic shock: Low-velocity and low-resistance phenotype with a flattened systolic peak and persistent diastolic forward flow.

### Correlations between SMA Doppler parameters and clinical severity markers

Correlations between superior mesenteric artery Doppler parameters and markers of clinical severity are presented in Table 3; Figure 4. RI and PI showed significant negative correlations with lactate, norepinephrine requirements, and SOFA score (all *p* < 0.001). PSV also correlated negatively with lactate, norepinephrine, SOFA score, and creatinine. BF showed weak correlations with all severity markers. No significant correlations were observed between EDV and any clinical parameters.

**Table 3:** Correlations between SMA Doppler parameters and markers of clinical severity.

Variables	Lactate		Pv-aCO_2_		MAP		Norepinephrine		SOFA		Creatinine	
	*ρ*	*P* value	ρ	*p* value	ρ	*p* value	ρ	*p* value	ρ	*p* value	ρ	*p* value
PSV	−0.49	<0.001	−0.42	<0.01	0.12	0.30	−0.44	<0.001	−0.61	<0.001	−0.40	<0.001
EDV	0.03	0.78	0.12	0.68	−0.20	0.10	0.17	0.15	0.12	0.31	0.04	0.15
TAMV	−0.28	0.02	−0.10	0.32	−0.21	0.07	−0.11	0.33	−0.29	0.02	−0.26	0.03
RI	−0.53	<0.001	−0.41	0.05	0.24	0.04	−0.59	<0.001	−0.64	<0.001	−0.40	<0.001
PI	−0.52	<0.001	−0.40	0.03	0.33	<0.01	−0.51	<0.001	−0.59	<0.001	−0.38	0.001
BF	−0.28	0.02	−0.10	0.43	−0.21	0.07	−0.15	0.19	−0.26	0.03	−0.27	0.02

Spearman's correlation coefficients (ρ) are presented. PSV, peak systolic velocity; EDV, end-diastolic velocity; TAMV, time-averaged mean velocity; RI, resistive index; PI, pulsatility index; BF, blood flow; Pv-aCO_2_, venous-to-arterial carbon dioxide difference; MAP, mean arterial pressure; SOFA, Sequential Organ Failure Assessment; Cr, creatinine.

**Figure 4:**
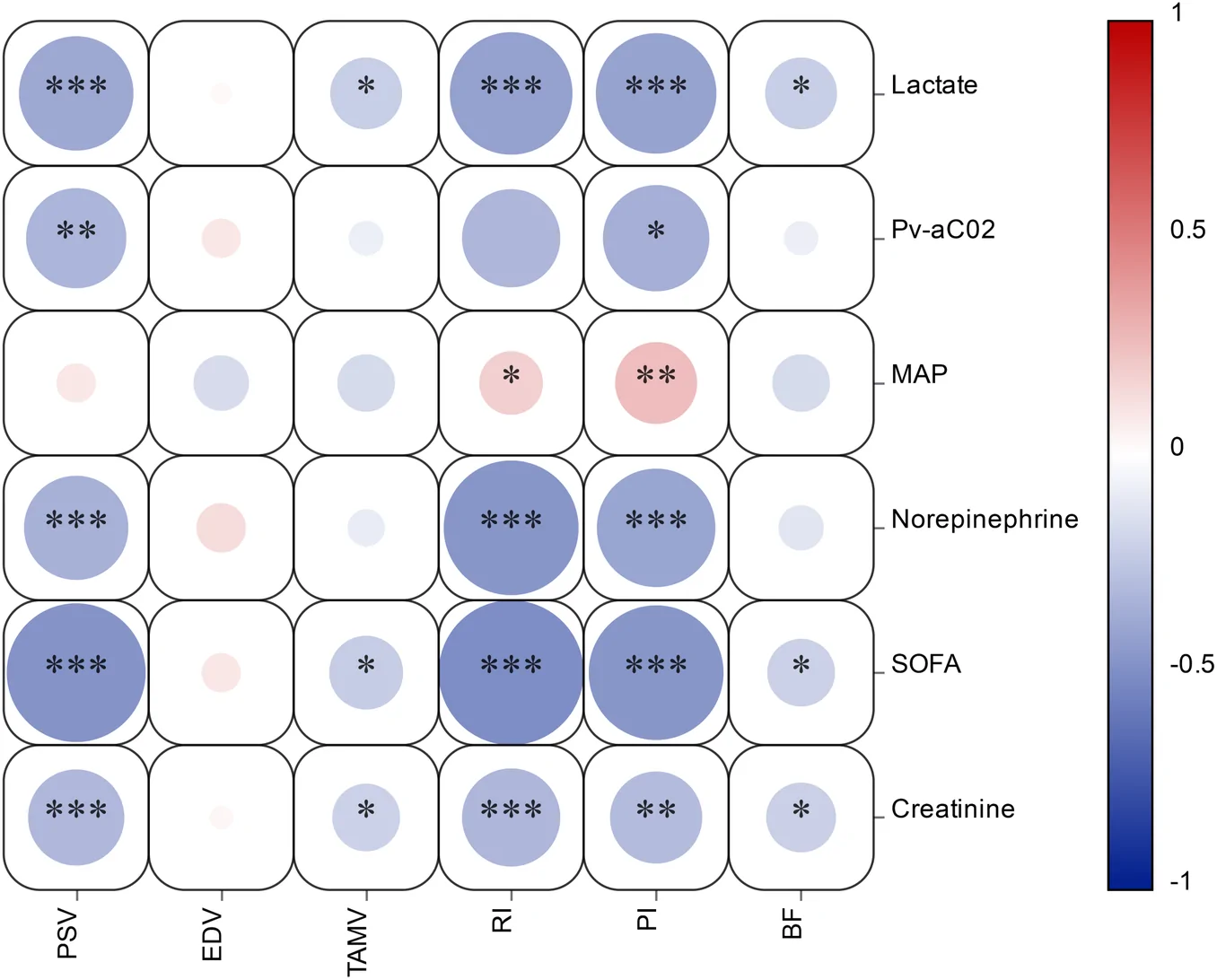
Correlation heatmap between SMA Doppler parameters and markers of clinical severity(*n* = 96). The Spearman correlation coefficients (*ρ*) are visualized by both circle size and color intensity. The color scale on the right indicates the strength and direction of the correlation: blue represents a negative correlation (*ρ* < 0), while red represents a positive correlation (*ρ* > 0). Asterisks denote statistical significance: * *p* < 0.05, ** *p* < 0.01, and *** *p* < 0.001.

### SMA-Doppler parameters across norepinephrine-dose subgroups

To further explore the relationship between vasopressor dose and mesenteric hemodynamics, patients were stratified into low, moderate, and high norepinephrine dose groups (Table 4; Figure 5). Age, sex, and BMI were comparable across subgroups. Severity scores, lactate, and Pv-aCO₂ increased significantly with norepinephrine dose (all *p* < 0.001), while MAP remained stable. Notably, RI and PI decreased progressively with increasing norepinephrine dose (both *p* < 0.001), whereas TAMV and BF showed no significant differences across dose strata. As detailed in Supplementary Table S1, patient distribution across the norepinephrine-equivalent dose strata demonstrates that patients with septic shock were distributed across the moderate-dose stratum and the high-dose stratum, whereas all patients with refractory septic shock were exclusively concentrated in the high-dose stratum (accounting for 23 of 30 patients in this tier, 76.7%). Therefore, the progressive reduction in RI and PI may largely reflect the underlying severity of sepsis and disease classification rather than a direct pharmacological dose-related vascular effect of norepinephrine.

**Table 4:** SMA Doppler parameters across norepinephrine dose subgroups.

Variables	No-NE (*n* = 25)	Low-dose (*n* = 16)	Moderate-dose (*n* = 25)	High-dose group (*n* = 30)	*p*-Value
Age (years)	71.00 (63.00, 77.00)	75.00 (65.00, 81.00)	77.00 (68.00, 83.00)	72.00 (60.00, 83.00)	0.387
BMI	23.22 ± 3.61	22.59 ± 3.60	21.92 ± 3.46	22.46 ± 4.60	0.797
APACHE II	20.00 (15.00, 25.00)	22.00 (19, 27.00)	26.00 (23.00, 35.00)	30.00 (26.25, 33.00)	<0.001
SOFA score	2.00 ± 1.00	4.00 ± 2.00	8.00 ± 2.00	10.00 ± 3.00	<0.001
Lactate(mmol/L)	1.00 (0.90, 1.30)	1.30 (1.00, 2.55)	2.75 (1.87, 3.93)	5.65 (3.62, 10.50)	<0.001
Pv-aCO_2_(mmHg)	5.00 (4.00, 6.00)	6.00 (4.00, 7.00)	6.00 (5.00, 8.00)	7.00 (6.00, 10.00)	<0.001
MAP (mmHg)	89 ± 10.00	88.00 ± 15.00	84.00 ± 14.00	83.00 ± 16.00	0.430
HR (bpm)	83 ± 14.00	100.00 ± 16.00	99.00 ± 21.00	108.00 ± 22.00	0.003
SMA Doppler Parameters					
PSV (cm/s)	125.89 ± 32.50	146.54 ± 48.71	139.91 ± 54.32	105.36 ± 48.45	0.004
EDV (cm/s)	20.73 ± 6.80	24.82 ± 6.70	27.80 ± 7.56	27.05 ± 8.53	0.013
RI	0.83 ± 0.03	0.83 ± 0.04	0.79 ± 0.05	0.72 ± 0.09	<0.001
PI	2.42 ± 0.42	2.44 ± 0.50	2.19 ± 0.59	1.72 ± 0.68	<0.001
TAMV (cm/s)	18.75 ± 5.75	21.55 ± 7.11	20.89 ± 6.53	19.58 ± 7.47	0.373
BF (ml/min)	385.55 ± 130.78	403.05 ± 152.60	392.34 ± 137.13	356.21 ± 147.68	0.625
CSA (cm^2^)	0.33 ± 0.07	0.32 ± 0.06	0.31 ± 0.08	0.30 ± 0.07	0.752

Patients were stratified by norepinephrine infusion rate at the exact time of the SMA Doppler examination: No-NE (no norepinephrine), low-dose (≤ 0.2 μg/kg/min), moderate-dose (> 0.2–0.5 μg/kg/min), and high-dose (> 0.5 μg/kg/min). APACHE II, Acute Physiology and Chronic Health Evaluation II; SOFA, Sequential Organ Failure Assessment; BMI, body mass index; Pv-aCO_2_, central venous-to-arterial carbon dioxide difference; MAP, mean arterial pressure; HR, heart rate; PSV, peak systolic velocity; EDV, end-diastolic velocity; TAMV, time-averaged mean velocity; RI, resistive index; PI, pulsatility index; BF, blood flow; CSA, cross-sectional area.

**Figure 5:**
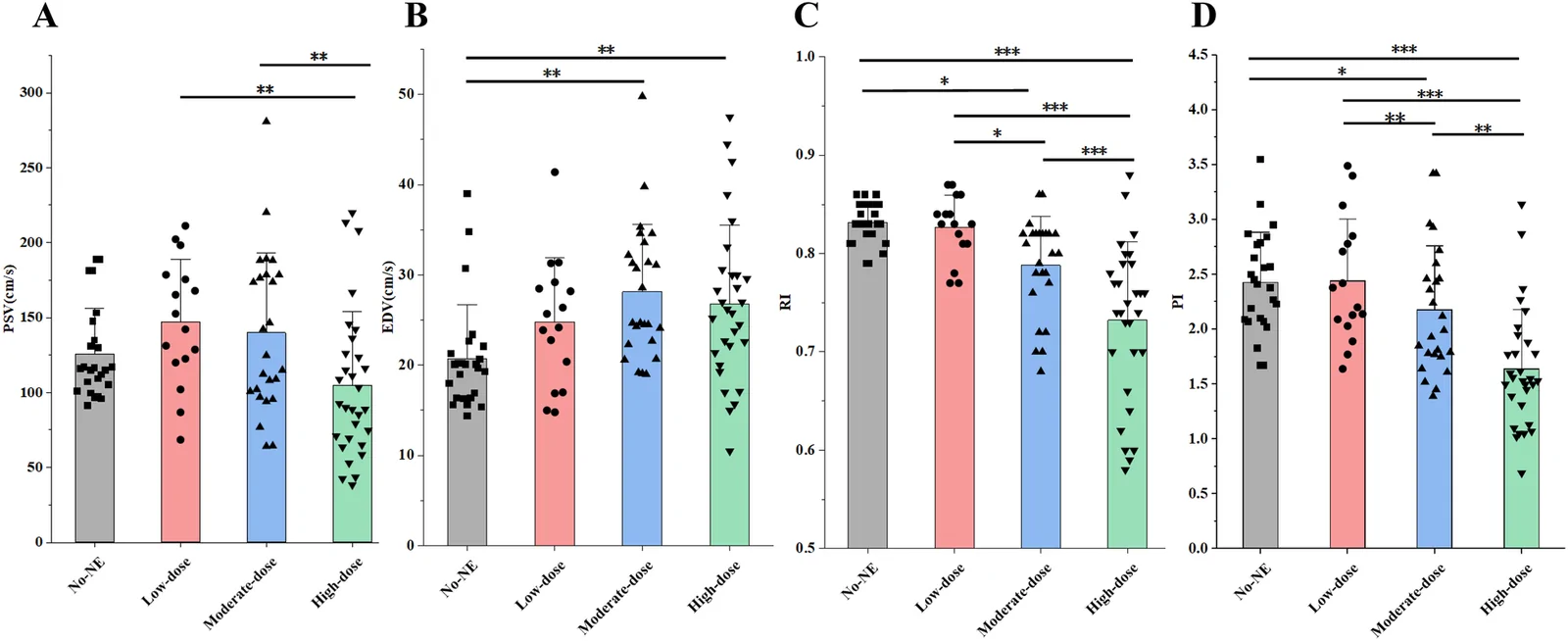
SMA Doppler parameters stratified by the norepinephrine infusion rate at the time of the SMA Doppler examination. Bar graphs with individual data points show mean ± standard deviation for **(A)** peak systolic velocity (PSV), **(B)** end-diastolic velocity (EDV), **(C)** resistive index (RI), and **(D)** pulsatility index (PI) across no norepinephrine (No-NE, *n* = 25), low-dose (≤0.2μg/kg/min, *n* = 16), moderate-dose (>0.2–0.5μg/kg/min, *n* = 25), and high-dose (>0.5μg/kg/min, *n* = 30) groups. One-way analysis of variance (ANOVA) followed by Tukey's *post-hoc* test with correction for multiple comparisons was used. *p* < 0.05, ** *p* < 0.01, *** *p* < 0.001.

### Exploratory discriminative indicators of refractory septic shock

To identify factors associated with refractory septic shock, univariable screening followed by a multivariable model using Firth's penalized logistic regression was performed (accounting for the modest sample size of *n* = 45) **(**Table 5). In this exploratory model, higher PSV was associated with lower odds of refractory septic shock (OR = 0.93, 95% CI: 0.88–0.98, *P* = 0.013). Similarly, preserved RI*10 showed an inverse association with refractory septic shock (OR = 0.12, 95% CI: 0.02–0.78, *P* = 0.026). Taken together, these findings suggest that PSV and RI may serve as preliminary exploratory discriminative markers for refractory septic shock.

**Table 5:** Univariate and multivariate firth penalized logistic regression analysis for refractory septic shock.

Variables	Univariate					Multivariate				
	β	S.E	Wald *χ*²	*P*	OR (95%CI)	β	S.E	Wald χ²	*P*	OR (95%CI)
PSV	−0.08	0.02	17.68	<.001	0.91 (0.86∼0.96)	−0.07	0.03	6.17	0.013	0.93 (0.88∼0.98)
EDV	−0.06	0.04	2.13	0.144	0.94 (0.87∼1.02)					
TAMV	−0.13	0.06	5.12	0.022	0.88 (0.79∼0.98)					
RI*10	−1.84	0.68	7.23	<.001	0.16 (0.04∼0.60)	−2.14	0.96	4.93	0.026	0.12 (0.02∼0.78)
PI	−1.42	0.83	2.89	0.089	0.24 (0.05∼1.24)					
BF	−0.02	0.01	7.23	0.007	0.98 (0.97∼0.99)					

OR, odds ratio; CI, confidence interval; PSV, peak systolic velocity; EDV, end-diastolic velocity; TAMV, time-averaged mean velocity; BF, blood flow; RI, resistive index; PI, pulsatility index.

### Discriminative performance of SMA Doppler parameters for refractory septic shock

ROC curve analyses were performed to evaluate the discriminative performance of SMA PSV and RI for refractory septic shock. The apparent AUC was 0.898 for PSV and 0.874 for RI; after bootstrap internal validation with 1,000 replicates, the optimism-corrected AUC was 0.858 for PSV and 0.823 for RI. The cut-off values were 89.45 cm/s for PSV and 0.745 for RI. At these cut-offs, PSV had a sensitivity of 75.0% and a specificity of 90.9%; RI had a sensitivity of 87.5% and a specificity of 68.2%. These cut-offs should be regarded as exploratory and require external validation before clinical application (Table 6; Figure 6).

**Table 6:** Comparison of the areas under the ROC curves for refractory septic shock.

Variables	Cut-off Value	Apparent AUC	Optimism-corrected AUC	Sensitivity	Specificity
PSV	89.45	0.898 (0.800–0.996)	0.858 (0.751–0.948)	0.750 (0.543–0.876)	0.909 (0.736–0.946)
RI	0.745	0.874 (0.777–0.971)	0.823 (0.692–0.929)	0.875 (0.766–0.985)	0.682 (0.473–0.836)

Bootstrap resampling with 1,000 replicates was performed to obtain optimism-corrected AUC values and 95% confidence intervals for sensitivity and specificity. AUC, area under the receiver operating characteristic curve; PSV, peak systolic velocity; RI, resistive index.

**Figure 6:**
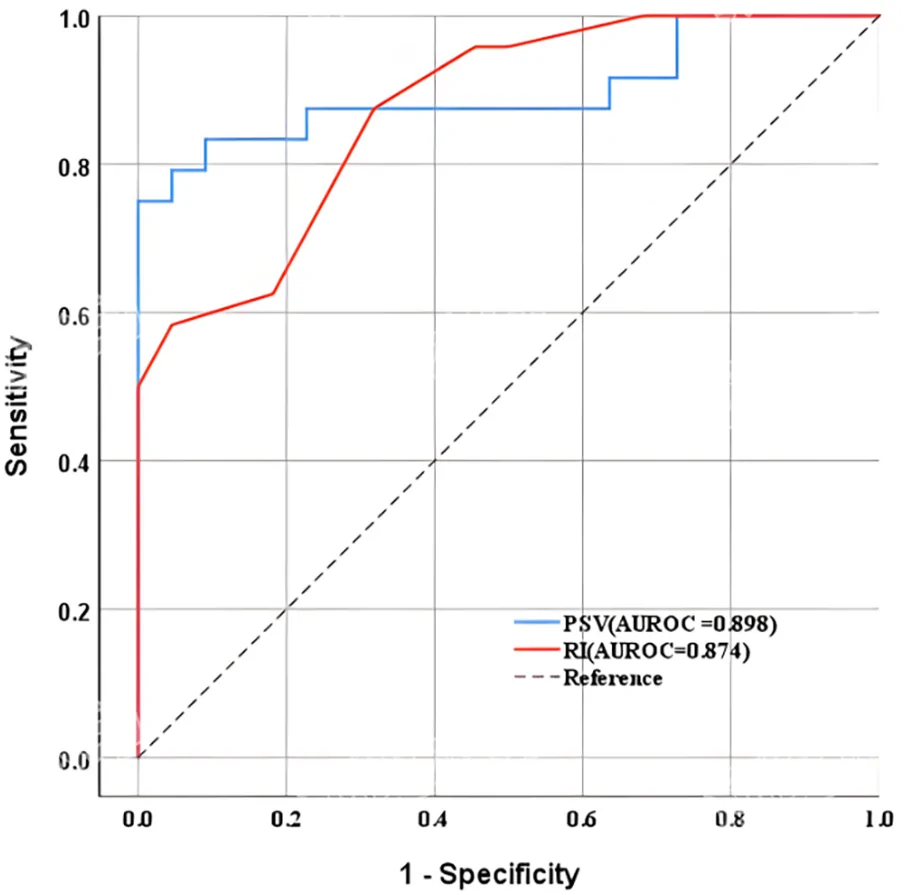
ROC curve analysis of the RI and PSV for discriminating refractory septic shock. The dashed line indicates the reference line.

### Paired comparison of SMA-Doppler parameters before and after successful resuscitation

Paired-sample analysis of 20 resuscitated shock patients revealed significant improvements in systemic hemodynamics, lactate levels and SOFA scores post-resuscitation (all *p* < 0.05) (Table 7). SMA-derived PSV and RI increased markedly (*p* < 0.001), while EDV declined significantly. TAMV and BF showed no change. Individual trajectories (Figure 7) showed changes in SMA Doppler waveform characteristics toward the pattern observed in less severe illness.

**Table 7:** Systemic hemodynamics and SMA Doppler parameters before and after successful resuscitation.

Characteristic	Pre-successful resuscitation (*n* = 20)	Post-successful resuscitation (*n* = 20)	*p*-Value
MAP (mmHg)	80.00 ± 14.00	87.00 ± 14.00	0.044
Lactate (mmol/L)	5.04 ± 2.50	1.80 ± 0.3	<0.001
Pv-aCO_2_(mmHg)	7.00 (5.00, 9.00)	5.00 (4.00, 6.00)	<0.001
NE (μg/kg/min)	0.60 (0.30, 1.00)	0.1 (0.00, 0.20)	<0.001
SOFA score	8.00 ± 2.00	3.00 ± 1.00	<0.001
SMA Doppler Parameters			
PSV (cm/s)	102.09 (89.35, 111.98)	127.80 (105.32, 143.55)	<0.001
EDV (cm/s)	24.85 (22.73, 29.90)	20.30 (18.25, 23.27)	<0.001
RI	0.75 (0.70, 0.78)	0.84 (0.82, 0.86)	<0.001
PI	1.69 (1.52, 1.84)	2.20（2.07, 2.64）	<0.001
TAMV (cm/s)	19.00 (16.20, 22.27)	19.20 (16.05, 22.62)	0.758
BF (ml/min)	372.90 (275.13, 438.48)	382.35 (320.44, 436.15)	0.561
CSA s(cm^2^)	0.32 (0.27, 0.37)	0.33 (0.27, 0.39)	0.874

Normality of within-patient differences was assessed using the Shapiro–Wilk test. Data are presented as mean ± SD for variables analyzed by paired *t*-test and as median (IQR) for variables analyzed by Wilcoxon signed-rank test. PSV, peak systolic velocity; EDV, end-diastolic velocity; TAMV, time-averaged mean velocity; RI, resistive index; PI, pulsatility index; BF, blood flow; Pv-aCO_2_, venous-to-arterial carbon dioxide difference; MAP, mean arterial pressure; SOFA, Sequential Organ Failure Assessment.

**Figure 7:**
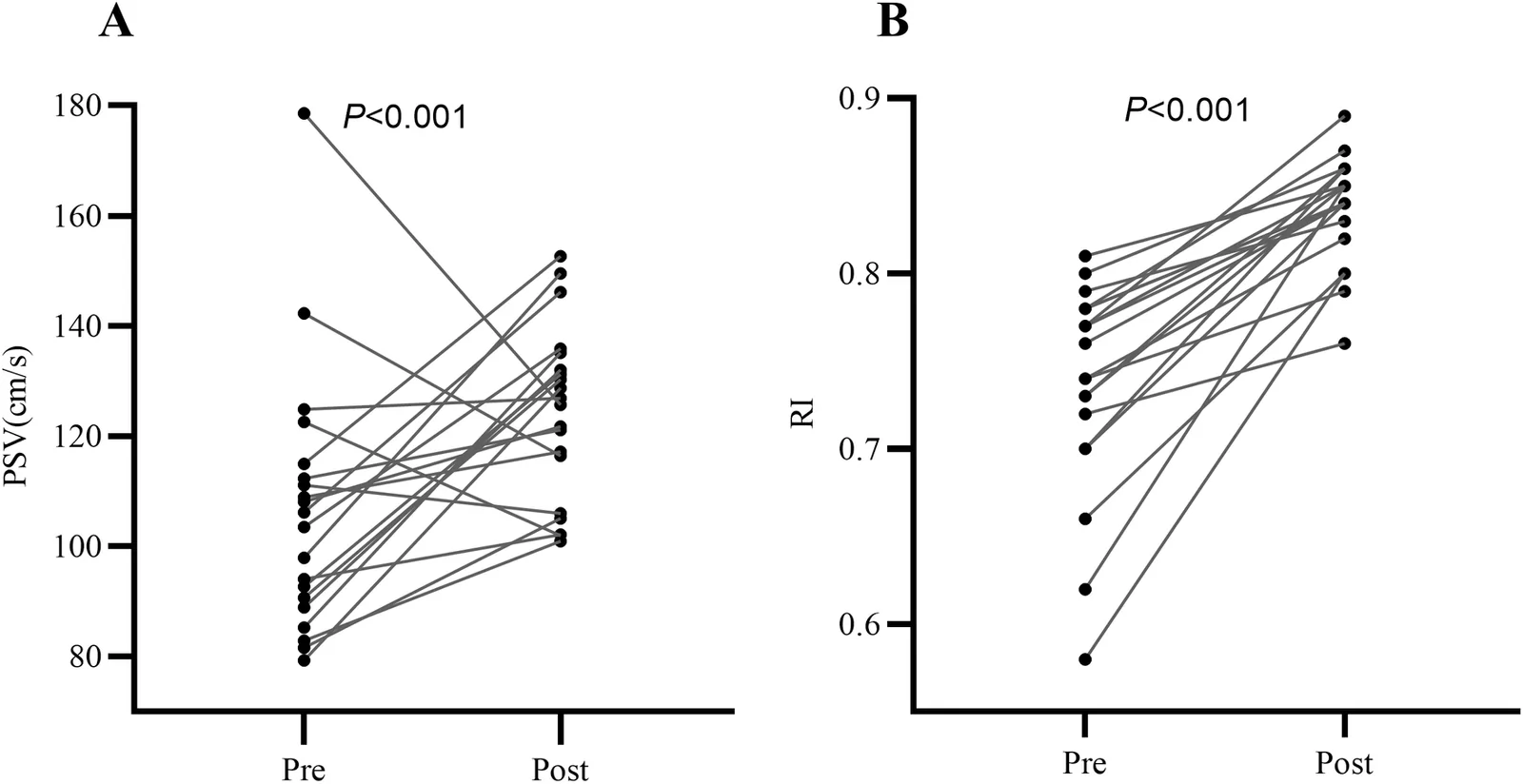
Paired individual changes in SMA Doppler parameters before and after successful resuscitation(*n* = 20). **(A)** Peak systolic velocity (PSV, cm/s); **(B)** Resistive index (RI). Each grey line connects pre- and post-resuscitation values from a single patient. Within-patient differences were assessed using the Wilcoxon signed-rank test. PSV and RI increased significantly after resuscitation (both *p* < 0.001.

## Discussion

In this study, we characterized SMA Doppler profiles across the sepsis severity spectrum. Sepsis was associated with a hyperdynamic SMA profile, whereas advanced shock, particularly refractory septic shock, was characterized by a low-velocity, low-resistance waveform phenotype. Lower PSV and RI showed exploratory discriminative potential for refractory septic shock. In the paired subgroup after successful resuscitation, PSV, RI, and PI increased, whereas TAMV and calculated SMA blood flow did not significantly change. These findings suggest that SMA Doppler may reflects dynamic changes in mesenteric arterial waveform characteristics across sepsis severity; however, these parameters should not be interpreted as direct markers of intestinal microcirculatory perfusion.

A defining finding of this study was the paradoxical reduction in SMA Doppler resistance indices during the progression from sepsis to refractory septic shock. RI decreased from 0.88 in sepsis to 0.72 in refractory septic shock, whereas PI declined from 2.97 to 1.50, indicating that the most critically ill patients exhibited a low-resistance SMA Doppler phenotype rather than the traditionally anticipated increase in downstream vascular resistance. This observation suggests that advanced septic shock is associated with a distinct alteration in mesenteric vascular regulation. However, RI and PI are composite Doppler waveform indices and should not be interpreted as direct measurements of vascular resistance. Their reduction may reflect complex changes involving systolic and diastolic velocity profiles, vascular impedance, arterial compliance, cardiac output, and downstream vascular conditions. Therefore, the biological significance of this low-resistance phenotype remains inferential. One potential explanation is that progressive septic shock may involve a transition from adaptive vasomotor regulation toward dysregulated vascular responsiveness. Early sepsis is frequently characterized by a hyperdynamic circulation with increased sympathetic activation and redistribution of blood flow to preserve vital organ function. In contrast, prolonged and severe inflammation may impair vascular reactivity through multiple interacting pathways, including excessive nitric oxide production, endothelial dysfunction, activation of ATP-sensitive potassium channels, altered adrenergic responsiveness, and the accumulation of endogenous vasodilatory mediators (20, 21). These mechanisms provide a plausible biological framework for altered vascular tone during advanced shock, but they were not directly evaluated in this study and therefore cannot be established as the cause of the observed Doppler phenotype.

Importantly, altered mesenteric vascular regulation should not be equated with improved regional perfusion. SMA Doppler provides information on regional arterial hemodynamics but does not directly measure intestinal microcirculation or tissue oxygenation. Experimental studies using complementary techniques have demonstrated substantial dissociation between mesenteric macrocirculatory flow and intestinal microcirculatory perfusion during sepsis. In a porcine peritonitis model (22), ultrasound transit-time flowmetry showed that during the hyperdynamic phase, cardiac output and SMA flow increased markedly, whereas jejunal and colonic muscularis microcirculatory blood flow remained substantially below baseline. Similarly, experimental bacteremia studies combining Doppler flowmetry, intravital microscopy, and laser Doppler fluxmetry demonstrated impaired small-bowel microvascular perfusion despite preserved SMA-level blood flow (23). Indocyanine green clearance (ICG) study in septic rats (24), using *in vivo* ICG, hepatic blood flow measurement, and cardiac output monitoring, showed depressed hepatic clearance despite increased hepatic blood flow and cardiac output. Collectively, these observations provide an important physiological framework for interpreting our findings: changes in SMA-level flow or waveform characteristics do not necessarily parallel tissue-level perfusion. Thus, the decline in SMA-RI observed in refractory septic shock should not be interpreted as evidence of improved intestinal perfusion; rather, it may represent a regional hemodynamic phenotype occurring within the broader context of impaired hemodynamic coherence.

The relationship between vasoactive therapy and the observed SMA phenotype also warrants careful consideration. In our cohort, lower RI and PI were associated with higher lactate concentrations, SOFA scores, and norepinephrine requirements. Although higher norepinephrine doses were associated with lower SMA resistive indices, this finding should not be interpreted as evidence of a direct norepinephrine dose–response effect. Norepinephrine requirements are intrinsically linked to shock severity, and all patients with refractory shock were in the high-dose norepinephrine group. Moreover, vasoactive agents have heterogeneous effects on the splanchnic circulation.

Norepinephrine has minimal effects on mesenteric blood flow in animal models: in a porcine study, medium-dose norepinephrine (0.4 μg/kg/min) increased renal and coronary blood flow without altering mesenteric flow (25). In contrast, in a porcine fecal peritonitis model, both epinephrine and norepinephrine reduced SMA flow and jejunal mucosal microcirculation. Dobutamine may improve splanchnic perfusion (26), while vasopressin constricts the splanchnic circulation (27). These observations indicate that the SMA Doppler phenotype cannot be attributed to norepinephrine exposure alone. Rather, it may reflect the integrated effects of shock severity, vascular responsiveness, cardiac performance, and the overall vasoactive regimen.

Several mechanical and hemodynamic variables may also confound SMA-Doppler indices. Elevated central venous pressure increases splanchnic venous afterload and may alter resistive-index measurements (28). Mechanical ventilation with PEEP reduces SMA flow and intestinal microcirculatory perfusion (29), and positive fluid balance can elevate intra-abdominal pressure, which negatively correlates with SMA blood flow (30). These factors were not systematically quantified at the time of Doppler assessment and therefore represent potential contributors to the observed phenotype. Future prospective studies incorporating these haemodynamic, ventilatory, and abdominal factors are needed to clarify their influence on SMA Doppler indices.

Exploratory discriminatory analyses suggest that SMA Doppler parameters may help identify refractory septic shock. In the acute-phase shock cohort, lower PSV and RI were associated with refractory shock in Firth's logistic regression, with relatively high apparent AUC on ROC analysis. Bootstrap internal validation (1,000 replicates) yielded optimism-corrected AUC of 0.858 for PSV and 0.823 for RI, lower than the apparent values but still indicating acceptable discrimination. However, these findings remain exploratory rather than validated discriminative markers; the small single-center sample and derivation/evaluation of cut-offs in the same cohort mean the cut-offs are still subject to residual optimism and should be considered exploratory. Future larger-scale prospective studies with external validation are warranted.

In the present study, SMA flow parameters (PSV, TAMV, and BF) increased in early-phase sepsis and declined progressively with worsening shock. Prior work shows that SMA flow is tightly coupled to cardiac output and cardiac index (10, 31). These reductions in SMA flow may therefore partly reflect global cardiac dysfunction due to sepsis-induced cardiomyopathy(SIC) (32) rather than isolated mesenteric vascular dysfunction alone. Because cardiac output was not measured, we cannot separate systemic cardiac performance from regional mesenteric hemodynamic alterations; therefore, PSV, TAMV, and BF should be regarded as exploratory discriminative markers, not definitive evidence of isolated mesenteric vascular dysfunction.

In our paired subgroup, following successful resuscitation, PSV and RI increased whereas TAMV and calculated SMA blood flow did not change significantly. This paired analysis is subject to inherent selection and survivorship bias because repeat measurements were obtained only in patients who achieved clinical recovery. These waveform changes do not establish restoration of mesenteric vascular tone or recovery of mesenteric perfusion. Rather, they indicate that SMA Doppler waveform characteristics changed toward the pattern observed in less severe illness or after clinical stabilization. These indices may serve as exploratory bedside markers of resuscitation response, but direct improvement in regional tissue perfusion was not measured. Given the limited sample size (*n* = 20), this finding remains exploratory.

The inverse association between SMA-RI and serum creatinine contrasts with the generally higher renal resistive index (RRI) observed with renal dysfunction and acute kidney injury (33, 34). Zheng et al. reported concordant elevations of SMA-RI and RRI in sepsis-associated intestinal–renal syndrome (11), Whereas our cohort showed declining SMA-RI with increasing shock severity despite renal dysfunction. Although indirect because RRI was not measured, this divergence raises the possibility that Doppler-derived vascular responses may be organ- and stage-specific during septic shock, warranting confirmation with simultaneous renal and mesenteric Doppler assessment.

This study has several limitations. First, its single-center design and relatively small sample size limit generalizability and increase the risk of residual confounding. Second, cardiac output was not systematically measured, preventing direct assessment of the contribution of global cardiac performance to SMA Doppler changes. Third, central venous pressure, intra-abdominal pressure, detailed fluid balance, ventilatory settings, and other determinants of splanchnic hemodynamics were not systematically incorporated. Fourth, the influence of the complete vasoactive regimen could not be fully separated from shock severity; in particular, the norepinephrine-dose analysis is inherently confounded by the refractory septic shock definition, because the high-dose threshold overlaps with the norepinephrine-equivalent dose criterion. Fifth, direct assessment of intestinal microcirculation or tissue perfusion was not performed; therefore, the relationship between SMA Doppler abnormalities and intestinal microcirculatory dysfunction remains uncertain. Sixth, the paired successful-resuscitation analysis is subject to selection and survivorship bias because repeat measurements were obtained only in patients who achieved clinical recovery. Seventh, pre-existing cardiovascular comorbidities and chronic beta-blocker use were not systematically collected or stratified, and a subset of patients received pituitrin as a rescue vasopressor because arginine vasopressin was unavailable. Finally, although bootstrap internal validation was performed, the exploratory ROC thresholds were derived and evaluated in the same cohort and have not undergone external validation; formal inter- and intra-observer reproducibility was not assessed.

## Conclusion

SMA Doppler profiles differed across the sepsis severity spectrum, with refractory septic shock characterized by a low-velocity, low-resistance waveform phenotype. Lower PSV and RI showed exploratory cross-sectional discrimination for refractory septic shock, but external validation is required. These indices should not be interpreted as direct measures of mesenteric vascular resistance or intestinal microcirculatory perfusion. SMA Doppler may complement systemic hemodynamic assessment by providing regional mesenteric information; prospective studies are warranted to validate its clinical utility and clarify its relationship with cardiac output and intestinal microcirculation.

## Data Availability

The raw data supporting the conclusions of this article will be made available by the authors, without undue reservation.
