# Supplementary material for: Superior mesenteric artery Doppler parameters across sepsis severity spectrum and their discriminative performance for refractory septic shock
Source: Front Med (Lausanne). 2026 Oct 27;13:1990309. doi: 10.3389/fmed.2026.1990309 (PMC13748340; doi:10.3389/fmed.2026.1990309)
Supplement: Supplementary file 1 [file Table1.docx]

Supplementary Material

# Supplementary Table

| **Norepinephrine dose strata** | **Total n** | **Sepsis n (%)** | **Septic shock** **n (%)** | **Refractory septic shock n (%)** |
| --- | --- | --- | --- | --- |
| No-NE | 25 | 3 (12.0) | 0 (0) | 0 (0) |
| Low dose (≤0.2μg/kg/min) | 16 | 16 (100.0) | 0 (0) | 0 (0) |
| Moderate dose (>0.2**–**0.5μg/kg/min) | 25 | 10 (40.0) | 15 (60.0) | 0 (0) |
| High dose (>0.5μg/kg/min) | 30 | 0 | 7 (23.3) | 23 (76.7) |

**Supplementary Table S1. Distribution of sepsis, septic shock and refractory septic shock patients across norepinephrine dose strata**

Data are presented as count (percentage) [n (%)]. Percentages are calculated based on the total number of patients within each dose stratum (*Total n*). Dose strata are defined based on the concurrent norepinephrine dose at the time of the SMA-Doppler examination.
